# Product-Form Stationary Distributions for Deficiency Zero Networks with Non-mass Action Kinetics

**DOI:** 10.1007/s11538-016-0220-y

**Published:** 2016-10-27

**Authors:** David F. Anderson, Simon L. Cotter

**Affiliations:** 1 Department of Mathematics, University of Wisconsin, Madison, WI USA; 2 Department of Mathematics, University of Manchester, Manchester, UK

**Keywords:** Product-form stationary distributions, Deficiency zero, Constrained averaging, Stochastically modeled reaction network

## Abstract

In many applications, for example when computing statistics of fast subsystems in a multiscale setting, we wish to find the stationary distributions of systems of continuous-time Markov chains. Here we present a class of models that appears naturally in certain averaging approaches whose stationary distributions can be computed explicitly. In particular, we study continuous-time Markov chain models for biochemical interaction systems with non-mass action kinetics whose network satisfies a certain constraint. Analogous with previous related results, the distributions can be written in product form.

## Introduction

Biological interaction systems are typically modeled in one of three ways. If the counts of the constituent species are high, then their concentrations are often modeled via a system of ordinary differential equations with state space ${\mathbb {R}}^d_{\ge 0}$, where $d>0$ is the number of species. If the counts are moderate (perhaps order $10^2$ or $10^3$), then they may be approximated by some form of continuous diffusion process (Gillespie 2000; Van Kampen 2007). If the counts are low, then the system is typically modeled stochastically as a continuous-time Markov chain in ${\mathbb {Z}}^d_{\ge 0}$ (Anderson and Kurtz 2011, 2015). We often want to understand the stationary behavior of the model under consideration. For deterministic models, understanding the stationary behavior usually entails characterizing the stable fixed points of the system, whereas for stochastic models, we require the calculation of the stationary distribution.

Stationary distributions are also useful in a multiscale setting, where the stationary statistics of a fast subsystem can be utilized in the approximation of the dynamics of the slow variables, which are typically of most interest. When an analytical form for the stationary distribution of the fast subsystem is not known, numerical approximations can be used. However, these computations are often expensive and part of an “inner loop,” typically making this calculation the rate-limiting step of the analysis. In the context of biochemical reaction networks, quasi-equilibrium (QE)-based approximations lead to fast subsystems which preserve mass action kinetics (Goutsias 2005; Janssen 1989; Thomas et al. 2012; Cao et al. 2005; Weinan et al. 2005). However, more recent improvements in stochastic averaging can lead to fast subsystems with non-mass action kinetics, and this observation was the motivation for the present work (Cotter 2016; Cotter and Erban 2016; Cotter et al. 2011).

One class of interaction networks that has been quite successfully analyzed, and that appears ubiquitously as fast subsystems, are those that are weakly reversible and have a deficiency of zero (see Appendix). For this class of models, and under the assumption of mass action kinetics, the fixed points of the deterministic models (Anderson 2011, 2008; Craciun 2015; Feinberg 1979, 1987; Gunawardena 2003) and the stationary distributions for the stochastic models, have been fully characterized (Anderson et al. 2010, 2015; Cappelletti and Wiuf 2016; Van Kampen 1976). In fact, it is the study of this class of networks that is largely responsible for the development of the field of chemical reaction network theory (Feinberg 1972, 1979; Gunawardena 2003; Horn 1972), a branch of applied mathematics in which the dynamical properties of the mathematical model are related to the structural properties of the interaction network.

In this article, we return to stochastically modeled interaction networks that are weakly reversible and have a deficiency of zero, though we consider propensity functions (also called intensity functions or rate functions) that are more general than mass action kinetics. However, we add a certain condition to the rates within the reaction network (see Assumption 1 below). Following Anderson et al. (2010), which was motivated by the work of Kelly (1979) who discovered the product-form stationary distribution of certain stochastically modeled queuing networks, we provide the form of the stationary distribution for this class of models. In particular, and in similarity with the main results of Anderson et al. (2010), in Theorem 2 we show that the distribution is of product form and that the key parameter of the distribution is a complex-balanced equilibrium value of an associated deterministically modeled system with mass action kinetics. The result of Anderson et al. (2010) can now be viewed as a special case of this new result.

The paper proceeds as follows: In Sect. 2, we introduce the formal mathematical model of interest. In Sect. 3, we provide the main theorem of this article, which characterizes the stationary distribution for the class of models of interest. In Sect. 4, we provide a series of examples which demonstrate the usefulness of the main result. Some brief concluding remarks are given in Sect. 5.

We assume throughout that the reader is familiar with terminology from chemical reaction network theory. However, we provide in Appendix all necessary terminology and results from this field that are used in the present work.

## Mathematical Model

We consider a system with *d* chemical species, $\{S_1,\dots ,S_d\}$, undergoing reactions which alter the state of the system. For concreteness, we suppose there are $K>0$ distinct reaction channels. For the *k*th reaction channel, we denote by $\nu _k, \nu _k' \in {\mathbb {Z}}^d_{\ge 0}$ the vectors representing the number of molecules of each species consumed and created in one instance of that reaction, respectively. Note that $\nu _k'-\nu _k\in {\mathbb {Z}}^d$ is the net change in the system due to one instance of the *k*th reaction. We associate each such $\nu _k$ and $\nu _k'$ with a linear combination of the species in which the coefficient of $S_i$ is $\nu _{ki}$, the *i*th element of $\nu _k$. For example, if $\nu _k = [1, \ 2]^\mathrm{T}$ for a system consisting of two species, then we associate with $\nu _k$ the linear combination $S_1 + 2S_2$. Under this association, each $\nu _k$ and $\nu _k'$ is termed a *complex* of the system. We denote any reaction by the notation $\nu _k \rightarrow \nu _k'$, where $\nu _k$ is the source, or reactant, complex and $\nu _k'$ is the product complex. We note that each complex may appear as both a source complex and a product complex in the system. The set of all complexes will be denoted by $\{\nu _k\}$.

### Definition 1

Let $\mathcal {S}= \{S_i\}$, $\mathcal {C}= \{\nu _k\},$ and $\mathcal {R}= \{\nu _k \rightarrow \nu _k'\}$ denote the sets of species, complexes, and reactions, respectively. The triple $\{\mathcal {S}, \mathcal {C}, \mathcal {R}\}$ is called a *chemical reaction network*.

Throughout, we assume that $\nu _k\ne \nu _k'$ for each $k\in \{1,\dots ,K\}$.

### Deterministic Model

The usual deterministic model for a chemical reaction network $\{\mathcal {S},\mathcal {C},\mathcal {R}\}$ assumes that the vector of concentrations for the species satisfies a differential equation of the form

1 $$\begin{aligned} \dot{x}(t) = \sum _{k=1}^K r_k(x(t)) (\nu _k' - \nu _k), \end{aligned}$$

where $r_k:{\mathbb {R}}^d_{\ge 0} \rightarrow {\mathbb {R}}_{\ge 0}$ is the (state-dependent) rate of the *k*th reaction channel. If we assume that each function $r_k$ satisfies deterministic mass action kinetics, then

$$\begin{aligned} r_k(x) = \kappa _k \prod _{i = 1}^d x_i^{\nu _{ki}}. \end{aligned}$$

#### Definition 2

An equilibrium value $c \in {\mathbb {R}}^d_{\ge 0}$ of (1) is said to be *complex balanced* if for each $\eta \in \mathcal {C}$,

$$\begin{aligned} \sum _{k: \nu _k=\eta } r_k(c) = \sum _{k:\nu _k' = \eta }r_k(c), \end{aligned}$$

where the sum on the left, respectively right, is over those reactions with $\eta$ as source complex, respectively product complex. In the special case of mass action kinetics, *c* is complex balanced if and only if

2 $$\begin{aligned} \sum _{k: \nu _k=\eta }\kappa _k \prod _{i = 1}^d c_i^{\nu _{ki}} = \sum _{k:\nu _k' = \eta } \kappa _k \prod _{i = 1}^d c_i^{\nu _{ki}}. \end{aligned}$$

### Stochastic Model, Previous Results, and Assumptions

The usual stochastic model for a reaction network $\{\mathcal {S}, \mathcal {C}, \mathcal {R}\}$ treats the system as a continuous-time Markov chain for which the rate of transition from state $x\in {\mathbb {Z}}^d_{\ge 0}$ to state $x +\nu _k'-\nu _k$ is $\lambda _k(x)$, where $\lambda _k:{\mathbb {Z}}^d_{\ge 0} \rightarrow {\mathbb {R}}_{\ge 0}$ is a suitably chosen intensity function (the intensity functions are also termed *propensity* functions in the literature). This stochastic process can be characterized in a variety of useful ways (Anderson and Kurtz 2011, 2015). For example, it is the stochastic process with state space ${\mathbb {Z}}^d_{\ge 0}$ and infinitesimal generator

$$\begin{aligned} Af(x) = \sum _{k=1}^K \lambda _k(x)(f(x+\nu _k'-\nu _k) - f(x)), \end{aligned}$$

where $f:{\mathbb {Z}}^d_{\ge 0} \rightarrow {\mathbb {R}}$. Kolmogorov’s forward equation for this model, termed the chemical master equation in the biology literature, is

3 $$\begin{aligned} \frac{\mathrm{d}}{\mathrm{d}t} p_\mu (t,x)= & {} \sum _{k=1}^K \lambda _k(x-\nu _k'+\nu _k) p_\mu (t,x-\nu _k' + \nu _k)\mathbbm {1}_{\{x-\nu _k' + \nu _k \ge 0\}}\nonumber \\&- \sum _{k =1}^K \lambda _k(x) p_\mu (t,x), \end{aligned}$$

4 $$\begin{aligned}= & {} A^*p_\mu (t,x)\mathbbm {1}_{\{x\ge 0\}}, \end{aligned}$$

where $p_\mu (t,x)$ is the probability the process is in state $x\in {\mathbb {Z}}^d_{\ge 0}$ at time $t\ge 0$, given an initial distribution of $\mu$, and $A^*$ is the adjoint of *A*. Note that (3) implies that a stationary distribution for the model, $\pi$, must satisfy

5 $$\begin{aligned} \sum _{k=1}^K \lambda _k(x-\nu _k'+\nu _k) \pi (x-\nu _k' + \nu _k) =\sum _{k =1}^K \lambda _k(x)\pi (x). \end{aligned}$$

Note also that (4) implies that $\pi$ is in the null space of $A^*$. Thus, and as is well known, finding $\pi$ reduces to solving $\pi A = 0$, with $\sum _x \pi (x)=1$, where *A* is reinterpreted as a generator matrix.

Of particular interest to us are the rate functions, $\lambda _k$. Under the assumption of stochastic mass action kinetics, we have

$$\begin{aligned} \lambda _k(x) =\kappa _k \prod _{i=1}^d \frac{x_i!}{(x_i - \nu _{ki})!} = \kappa _k \prod _{i = 1}^d \prod _{j=0}^{\nu _{ki}-1} (x_i-j), \end{aligned}$$

where $\kappa _k>0$ are the *reaction rate constants*. Here and throughout, we interpret any product of the form $\prod _{i = 0}^{-1} a_i$ to be equal to one. In Anderson et al. (2010), the authors considered a class of stochastically modeled reaction networks with mass action kinetics (those with a deficiency of zero and are weakly reversible, see Appendix) and characterized their stationary distributions as products of Poisson distributions. However, they did not just consider mass action kinetics in Anderson et al. (2010), but any kinetics satisfying the functional form

6 $$\begin{aligned} \lambda _k(x) = \kappa _k \prod _{i = 1}^d \prod _{j=0}^{\nu _{ki}-1}\theta _i(x_i-j), \end{aligned}$$

so long as $\theta _i(0)=0$ for each *i*. In particular, they proved the following.

#### Theorem 1

(Anderson et al. 2010) Let $\{\mathcal {S},\mathcal {C},\mathcal {R}\}$ be a reaction network, and let $(\kappa _1,\dots ,\kappa _k)$ be a choice of positive rate constants. Suppose that modeled deterministically with mass action kinetics and rate constants $\kappa _k$, the system is complex balanced with complex-balanced equilibrium $c \in {\mathbb {R}}^d_{>0}$. Then, the stochastically modeled system with intensity functions (6) admits the invariant measure

7 $$\begin{aligned} \pi (x) = \prod _{i = 1}^d \frac{c_i^{x_i}}{ \prod _{j=0}^{ x_i-1} \theta _i(x_i-j)}. \end{aligned}$$

The measure $\pi$ can be normalized to a stationary distribution so long as it is summable.

The connection between weakly reversible, deficiency zero networks, and complex-balanced equilibria is given in Appendix.

In this paper, we generalize Theorem 1 by showing how to find the stationary distributions for models with rates that do not seem to satisfy the form (6). However, we add an assumption pertaining to the form of the rates within the reaction network, which we describe now. We begin by partitioning the set of species into two sets, $\mathcal {S} = \mathcal {S}_1\cup \mathcal {S}_2$. We will say that $S_i \in \mathcal {S}_2$ if $\alpha _i = \gcd \{\nu _{1i}, \nu _{2i}, \ldots , \nu _{Ki} \} >1$. Otherwise, we say that $S_i \in \mathcal {S}_1$, noting that $\alpha _i = 1$. We now assume that the intensity functions for the reaction network are of the form

8 $$\begin{aligned} \lambda _k(x) = \kappa _k \prod _{i = 1}^d \prod _{j=0}^{\frac{\nu _{ki}}{\alpha _i} - 1} \theta _i(x_i - j\alpha _i), \end{aligned}$$

where $\kappa _k>0$ and $\theta _i(x_i) = 0$ if and only if $x_i\le \alpha _i-1$.

#### Assumption 1

A stochastically modeled reaction network satisfies this assumption if it satisfies the partition described above and has intensity functions of the form (8).

We provide an example to clarify the notation.

#### Example 1

Consider the stochastically modeled system with reaction network9where the intensity functions are placed next to the reaction arrows. Here $S_1\in \mathcal {S}_2$, with $\alpha _1 = 2$, and $S_2,S_3 \in \mathcal {S}_1$. The assumption (8) then supposes that for appropriate functions $\theta _1,\theta _2,\theta _3:{\mathbb {Z}}_{\ge 0} \rightarrow {\mathbb {R}}_{\ge 0}$, we have

$$\begin{aligned} \lambda _1(x)&= \kappa _1 \theta _1(x_1)\\ \lambda _2(x)&= \kappa _2 \theta _2(x_2)\\ \lambda _3(x)&= \kappa _3\theta _1(x_1)\theta _1(x_1-2) \theta _2(x_2)\theta _2(x_2-1)\\ \lambda _4(x)&= \kappa _4 \theta _3(x_3). \end{aligned}$$

For example, valid choices include $\theta _1(x_1) = x_1(x_1-1)+1(x_1\ge 2)$, $\theta _2(x_2) = x_2$, and $\theta _3(x_3) = \frac{x_3}{1+x_3}$, in which case the form for the stationary distribution does not follow immediately from Theorem 1.

## Main Result

Here we state and prove our main result.

### Theorem 2

Let $\{\mathcal {S}_1\cup \mathcal {S}_2,\mathcal {C},\mathcal {R}\}$ be a reaction network satisfying Assumption 1, and let $(\kappa _1,\dots ,\kappa _K)$ be a choice of positive rate constants. Suppose that modeled deterministically with mass action kinetics and rate constants $\kappa _k$ the system is complex balanced with complex-balanced equilibrium $c \in {\mathbb {R}}^d_{>0}$. Then the stochastically modeled system with intensity functions (8) admits the invariant measure

10 $$\begin{aligned} \pi (x) = \prod _{i = 1}^d \frac{c_i^{x_i}}{ \prod _{j=0}^{\lfloor x_i/\alpha _i\rfloor -1} \theta _i(x_i-j\alpha _i)}. \end{aligned}$$

The measure $\pi$ can be normalized to a stationary distribution so long as it is summable.

Note that the theorem applies to models with reaction networks satisfying Assumption 1 and that are weakly reversible and have a deficiency of zero. See Appendix.

### Proof

First note that if $\mathcal {S}_2 = \emptyset$, then Theorem 2 is the same as Theorem 1 and there is nothing to show. Thus, we suppose $\mathcal {S}_2 \ne \emptyset$.

The proof proceeds in the following manner. First, for each $S_i \in \mathcal {S}_2$ we will demonstrate the existence of a function $\varphi _i:{\mathbb {Z}}_{\ge 0} \rightarrow {\mathbb {R}}_{\ge 0}$ for which

11 $$\begin{aligned} \theta _i(x_i) = \prod _{\ell =0}^{\alpha _i-1} \varphi _i(x_i-\ell ) = \varphi _i(x_i)\cdots \varphi _i(x_i-\alpha _i+1). \end{aligned}$$

Next, we will apply Theorem 1 and prove that the resulting distribution is indeed given by (10).

Let $S_i \in \mathcal {S}_2$. We begin by setting

$$\begin{aligned} \varphi _i(0)=0, \quad \text {and}\quad \varphi _i(z) = 1, \text { for } 1\le z \le \alpha _i-1. \end{aligned}$$

For $z \ge \alpha _i$ an integer, we may define $\varphi _i$ recursively via the formula

12 $$\begin{aligned} \varphi _i(z) = \frac{\theta _i(z)}{\varphi _i(z-1)\cdots \varphi _i(z-\alpha _i+1)}. \end{aligned}$$

Note that $\varphi _i$ is a well-defined function since $\theta _i(z) > 0$ for each $z \ge \alpha _i$ by assumption. It is clear that (11) is satisfied with this choice of $\varphi _i$.

For $x\in {\mathbb {Z}}^d_{\ge 0}$, we may now write

$$\begin{aligned} \lambda _k(x)&= \kappa _k \prod _{i = 1}^d \prod _{j=0}^{\frac{\nu _{ki}}{\alpha _i} - 1} \theta _i(x_i - j\alpha _i) = \kappa _k \prod _{i = 1}^d \prod _{j=0}^{\frac{\nu _{ki}}{\alpha _i} - 1} \prod _{\ell =0}^{\alpha _i-1} \varphi _i(x_i-j \alpha _i - \ell )\\&=\kappa _k \prod _{i=1}^d \prod _{b=0}^{\nu _{ki}-1} \varphi _i(x_i - b). \end{aligned}$$

Hence, we may apply Theorem 1 and conclude that

$$\begin{aligned} \pi (x) = \prod _{i = 1}^d \frac{c_i^{x_i}}{ \prod _{j=0}^{ x_i-1} \varphi _i(x_i-j)} \end{aligned}$$

is an invariant measure for the system, where *c* is a complex-balanced fixed point for the deterministic system. It remains to show that

13 $$\begin{aligned} \prod _{j=0}^{ x_i-1} \varphi _i(x_i-j) = \prod _{j=0}^{\lfloor x_i/\alpha _i\rfloor -1} \theta _i(x_i-j\alpha _i). \end{aligned}$$

First note that if $x_i < \alpha _i$, then both sides of (13) are equal to one. For the time being, assume that $\alpha _i \le x_i< 2\alpha _i$. Under this assumption, the right-hand side of (13) is

$$\begin{aligned} \theta _i(x_i) = \varphi _i(x_i)\cdots \varphi _i(x_i - \alpha _i + 1) = \varphi _i(x_i)\cdots \varphi _i(\alpha _i ), \end{aligned}$$

where in the final equality we used that $\varphi _i(\ell ) = 1$ for $1 \le \ell < \alpha _i$ (and that $x_i - \alpha _i < \alpha _i$), and the left-hand side is

$$\begin{aligned} \varphi _i(x_i)\cdots \varphi _i(1) = \varphi _i(x_i)\cdots \varphi (\alpha _i), \end{aligned}$$

where we again used that $\varphi _i(\ell ) = 1$ when $1\le \ell < \alpha _i$. Hence, (13) is verified when $x_i <2 \alpha _i$.

We will now prove that (13) holds in general by induction. We suppose that (13) holds for all $z \le x_i$, where $x_i \ge 2\alpha _i-1$, and will show it to hold at $x_i + 1$. Using (12), the left-hand side of (13) evaluated at $x_i+1$ is

$$\begin{aligned}&\prod _{j=0}^{(x_i+1)-1}\varphi _i(x_i+1-j) \\&\qquad = \prod _{j=1}^{x_i+1} \varphi _i(j)=\varphi _i(x_i+1)\cdots \varphi _i(x_i+1-\alpha _i + 1) \prod _{j= 1}^{x_i+1 - \alpha _i} \varphi _i(j)\\&\qquad = \theta _i(x_i+1)\prod _{j= 1}^{x_i+1 - \alpha _i} \varphi _i(j), \end{aligned}$$

where the final equality is an application of (12) with $x_i+1$ in place of the variable *z*. Continuing, we have

14 $$\begin{aligned}&\theta _i(x_i+1)\prod _{j= 1}^{x_i+1 - \alpha _i} \varphi _i(j)\nonumber \\&\quad =\theta _i(x_i+1) \prod _{j=0}^{x_i+1-\alpha _i - 1} \varphi _i(x_i+1-\alpha _i - j)\qquad \quad \text {(by a rearrangement)}\nonumber \\&\quad =\theta _i(x_i+1) \prod _{j=0}^{\lfloor (x_i+1-\alpha _i)/\alpha _i\rfloor -1} \theta _i((x_i+1-\alpha _i)-j\alpha _i)\quad \text {(by the inductive hypothesis)}\nonumber \\&\quad = \theta _i(x_i+1) \prod _{j=0}^{\lfloor (x_i+1)/\alpha _i\rfloor -2} \theta _i(x_i+1-(j+1)\alpha _i)\nonumber \\&\quad = \theta _i(x_i+1) \prod _{j=1}^{\lfloor (x_i+1)/\alpha _i\rfloor -1} \theta _i(x_i+1-j\alpha _i)\nonumber \\&\quad = \prod _{j=0}^{\lfloor (x_i+1)/\alpha _i\rfloor -1} \theta _i(x_i+1-j\alpha _i), \end{aligned}$$

where the final equalities are straightforward. Note that (14) is the right-hand side of (13) evaluated at $x_i+1$, and so the proof is complete. $\square$

## Examples

### Example 1: Motivating Example

First, we consider a motivating example arising from model reduction, through constrained averaging (Cotter 2016; Cotter and Erban 2016; Cotter et al. 2011), of the following system:15where the intensity functions are placed next to the reaction arrows. Note that the intensities of all the reactions follow mass action kinetics. We consider this system in a parameter regime where the reversible dimerization reactions $2S_1 \rightleftharpoons S_2$ are occurring more frequently than the production of $S_2$ and the degradation of $S_1$. Both $S_1$ and $S_2$ are changed by the fast reactions, but the quantity $S = S_1 + 2S_2$ is invariant with respect to the fast reactions, and as such is the slow variable in this system. We wish to reduce the dynamics of this system to a model only concerned with the possible changes in *S*:16where $\bar{\lambda }_3(s)$ and $\bar{\lambda }_4(s)$ are the effective rates of the system.

Using the QE approximation (QEA), $\bar{\lambda }_3(s)=\kappa _3$ and $\bar{\lambda }_4(s)=\kappa _4 \mathbb {E}_{\pi _\mathrm{QEA}(s)}[X_1]$, where $\pi _\mathrm{QEA}(s)$ is the stationary distribution for the system17under the assumption that $X_1(0) + 2X_2(0) = s$. Since the system (17) satisfies the necessary conditions of the results of Anderson et al. (2010) (weak reversibility and deficiency of zero), the invariant distribution $\pi _\mathrm{QEA}(s)$ is known exactly.

In comparison, the constrained approach requires us to find the invariant distribution $\pi _\mathrm{Con}$ of the following system:18subject to $X_1(0)+2X_2(0)=s$. Readers interested in seeing how this is derived should refer to Cotter (2016). This network is weakly reversible and has a deficiency of zero. However, the form of the rates in this system does not satisfy the conditions specified in Anderson et al. (2010). In the context of constrained averaging, this lack of a closed form for the stationary distribution would result in the need for some form of approximation of the stationary distribution. There are two common methods utilized for performing this approximation. One possibility would be to perform exhaustive stochastic simulation of the system (18). Another option involves finding the distribution by finding the null space of the adjoint of the generator (see the discussion in and around (5)). However, as the state space of (18) will typically be huge, the latter method often involves truncating the state space and approximating the actual distribution with that of the stationary distribution of the truncated system (Cotter 2016). Both approaches will lead to approximation errors and varying amounts of computational cost. However, note that the system (18) does satisfy Assumption 1, with $\alpha _1 = 2$ and $\alpha _2 = 1$. We denote the rate of dimerization by $\lambda _D$ and its reverse by $\lambda _{-D}$. Therefore,

$$\begin{aligned} \lambda _D(x)= & {} k_1x_1(x_1-1) + k_3\mathbbm {1}_{\{x_1>1\}},\\= & {} k_1\left( x_1(x_1-1) + \frac{k_3}{k_1}\mathbbm {1}_{\{x_1>1\}} \right) ,\\= & {} k_1 \theta _1(x_1), \end{aligned}$$

with $\theta _1$ defined in the final equality. The form of the rate of the reverse reaction is much simpler and is given by

$$\begin{aligned} \lambda _{-D}(x)= & {} (k_2+k_4)x_2 = (k_2 + k_4)\theta _2(x), \end{aligned}$$

which defines $$\theta _2$$.

By Theorem 2, we can write down the stationary distribution of this system. The complex-balanced equilibrium of the associated deterministically modeled system with $c_1+2c_2=1$ is given by $(c_1, c_2) = \left( \frac{ \sqrt{(k_2+k_4)(k_2 + 8k_1 + k_4)} - k_2 - k_4}{4k_1}, \frac{1 - c_1}{2} \right)$. Then by Theorem 2, and by recalling that all states $(x_1,x_2)$ in the domain satisfy $s = x_1 + 2x_2$, the stationary distribution for $S_2$ is given by

19 $$\begin{aligned} \pi _\mathrm{{Con}}(x_2) = \frac{1}{\Gamma _\mathrm{{Con}}} \frac{c_1^{(s-2x_2)}}{\prod _{j=0}^{\lfloor (s-2x_2)/2\rfloor -1} \left( (s-2x_2-2j)(s-2x_2 -2j-1) + \frac{k_3}{k_1} \right) } \frac{c_2^{x_2}}{x_2!},\nonumber \\ \end{aligned}$$

where $\Gamma _\mathrm{{Con}}$ is a normalization constant and *s* is the conserved quantity. Note that the indicator function in $\theta _1$ (in the denominator) has disappeared since it is always equal to one over the domain of the product.

We can compare (19) with the distribution of (17), which arises from the QEA, and also with the distribution of the full system (15) conditioned on $S_1 + 2S_2 =s$ (which can be approximated by finding the null space of the adjoint of the generator of the full system on the truncated domain). First, we consider the QEA approximation. The invariant distribution of the fast subsystem (17) can be found using Theorem 1 and is given by

20 $$\begin{aligned} \pi _\mathrm{{QEA}}(x) = \frac{1}{\Gamma _\mathrm{{QEA}}} \frac{d_1^{s-2x_2}}{(s-2x_2)!} \frac{d_2^{x_2}}{x_2!}, \end{aligned}$$

where $(d_1, d_2) = \left( \frac{\sqrt{k_4(8k_1 + k_4)}-k_4}{4k_1},\frac{1- d_1}{2} \right)$ is the complex-balanced equilibrium for this system satisfying $d_1+2d_2=1$ and $\Gamma _\mathrm{{QEA}}$ is a normalizing constant.

Since the full system (15) does not have a deficiency of zero, we are not able to find its invariant distribution directly. However, by truncating the state space appropriately, we are able to approximate the full distribution by constructing the generator on this truncated state space and finding the null space of the adjoint.

Once we have approximated the null space of the truncated generator, we can find the approximation of $\mathbb {P}(X_2 = x_2|X_1 + 2X_2=s)$ by taking the probabilities of all states with $x_1+2x_2 = s$ and renormalizing. In what follows, we truncated the domain of the generator to $x \in \{0,1,\ldots ,1000\} \times \{0,1,\ldots ,500\}$.

We consider the system (15) with parameters given by:

21 $$\begin{aligned} k_1 = 1, \qquad k_2 = 100, \qquad k_3 = 1500, \qquad k_4 = 30. \end{aligned}$$

Note that it is not obvious from these rates that the reactions with rates $k_3$ and $k_4$ are in fact the slow reactions in this system. The invariant density is largely concentrated in a small region centered close to the point $x = (99,114)$. By using the approximation of the invariant density that we have computed on the truncated domain, we can compute the expected ratio between occurrences of the fast reactions with rates $k_1$ and $k_2$ with the slow reactions with rates $k_3$ and $k_4$. For this choice of parameters, the expected proportion of the total reactions which are fast reactions (dimerization/disassociation) is $82.68\,\%$. This indicates a difference in timescales between these reactions, but the difference is not particularly stark, and as such we would expect there to be significant error in any approximation relying on the QEA.

**Fig. 1:**
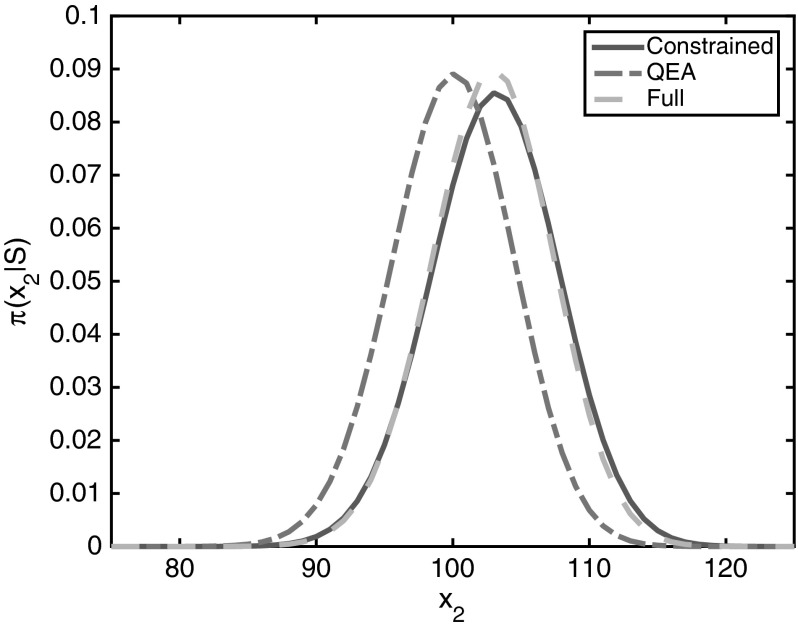
(Color figure online) Approximations of the distribution $\mathbb {P}(X_2=x_2|X_1 + 2X_2=300)$ for the system (15) with parameters given by (21) using constrained averaging, QEA averaging, and through approximation of the invariant distribution of the full system on $x \in \{0,1,\ldots ,1000\} \times \{0,1,\ldots ,500\}$.

Figure 1 shows the three approximations of the distribution $\mathbb {P}(X_2=x_2|X_1+2X_2=300)$ for the system (15) with parameters given by (21). The constrained and QEA approximations are computed using (19) and (20), respectively, with the normalizing constants computed numerically. As would be expected in this parameter regime, the constrained approximation is far more accurate than the QEA.

We can quantify the accuracy of each of the approximations by computing the relative $l^2$ differences with the distribution computed using the full generator on the truncated domain. This relative difference was $4.464 \times 10^{-1}$ for the QEA, in comparison with $5.2337\times 10^{-2}$ for the constrained approximation. This demonstrates the improvement in approximation that can be achieved by using constrained averaging, and which motivates the need for results like Theorem 2 which take non-mass action kinetics into account.

### Example 2: Dimerization

We begin by considering a model consisting of only proteins, denoted *P*, and dimers, denoted *D*. We suppose there are two mechanisms by which the proteins are dimerized: random interactions between the protein molecules, and via a catalyst. The rate at which random interactions lead to the formation of dimers can be taken to be of mass action form. Assuming the concentrations are such that the catalyst is acting at capacity, the rate of formation of the dimers due to the catalyst can be faithfully modeled as a constant (so long as there are at least two proteins to make the reaction happen). Thus, letting $x_p$ and $x_d$ denote the numbers of proteins and dimers in the model, respectively, the reaction system can be represented as22where $\kappa _{p\rightarrow d}$, $\kappa _{d\rightarrow p}$ and $\rho$ are given parameters. Note that after an obvious change of variables the stationary distribution of this particular model is provided in (19).

The reactions in (22) are typically a subset of the reactions in a larger system. For example, the actual model of interest may be23where *M* represents an mRNA molecule. This is a standard model for dimer production. Depending upon the relevant timescales in the system, we may want to take $d_D = \kappa _d = 0$. If the reactions $2P\rightleftharpoons D$ are appreciably faster than those of (23), then an obvious path simulation strategy presents itself in the mold of the slow-scale SSA (Cao et al. 2005) (i.e., stochastic averaging):

1. for the current values of *P* and *D*, find the stationary distribution of the fast subsystem analytically,
2. determine the effective rates of the reduced model 24 where the expectations are with respect to the distribution found in step 1.
3. simulate forward in time using the stochastic simulation algorithm (Gillespie 1976) or the next reaction method (Anderson 2007; Gibson and Bruck 2000), and return to step 1.

Being able to analytically calculate the stationary distribution in step 1 allows us to bypass the need to numerically approximate the stationary distribution, as is commonly done (Weinan et al. 2005; Weinan and Vanden-Eijnden 2007).

### Example 3

In Sects. 4.1 and 4.2, we applied Theorem 2 on the common motif $2S_1 \rightleftharpoons S_2$. In this example, we present another common motif, $2S \rightleftharpoons \emptyset$, for which Theorem 2 is also useful. As opposed to the specific models we considered in the previous examples, here we present a more general framework in which the specific form of the propensity function for the reaction $2S \rightarrow \emptyset$ is arbitrary. This situation is common when undertaking certain types of averaging arguments (Cotter 2016).

Let us suppose that the effective dynamics of a slow variable in a larger system can be modeled aswhere $k^b$ and $k_1^d$ are positive constants and where $\mathbb {E}(f_1,\dots ,f_r|S=s)$ is a conditional expectation of the fast variables $f_1,\dots ,f_r$, conditioned on $S=s$. The conditional expectation could in general be highly nonlinear, and not of the form (6) required by Theorem 1. Supposing that $\mathbb {E}(f_1,\dots ,f_r|S=s) = 0$ if $s\in \{0,1\}$, Theorem 2 says that, up to a normalization constant, the invariant distribution of *S* is given by

$$\begin{aligned} \pi (s) \propto \frac{ \left( \frac{k^b}{k^d_1} \right) ^s}{ \prod _{j=0}^{\lfloor s/2\rfloor -1} \mathbb {E}\left( f_1,\dots ,f_r|S=s-2j\right) }. \end{aligned}$$

In practice, the normalization constant could be approximated by summing over an appropriate domain.

### Example 4

This example will demonstrate the difficulties that can arise when a reaction is added that involves a species in the set $\mathcal {S}_2$ with multiplicity not equal to $\alpha _i$. In particular, consider the system25where

$$\begin{aligned} \lambda _1(x) = \kappa _1 \theta _1(x_1), \quad \lambda _2(x) = \kappa _2 , \quad \lambda _3(x) = \kappa _3 \varphi (x_1),\quad \lambda _4(x) = \kappa _4x_2, \end{aligned}$$

where $\theta _1(x_1) = \left( \mathbbm {1}_{\{x_1>1\}}(10 + x_1 + 6\sin (\pi x_1/5)) \right)$. Note that we have a non-mass action kinetics rate $\lambda _1$ for the reaction $2S_1 \rightarrow \emptyset$. There is another reaction involving $S_1$, but the amount of $S_1$ molecules involved in this reaction is not a multiple of 2. This means that we cannot apply the result of Theorem 2 to this system unless $\varphi$ satisfies the recurrence relation detailed in the proof of Theorem 2. In particular, it must satisfy $\varphi (x_1)\varphi (x_1-1) = \theta _1(x_1)$, or

$$\begin{aligned} \varphi (0)= & {} 0, \\ \varphi (1)= & {} C \in \mathbb {R}_{>0},\\ \varphi (n)= & {} \frac{\theta _1(n)}{\varphi (n-1)}. \end{aligned}$$

This recurrence relation defines a unique function $\varphi :\mathbb {Z}_{\ge 0} \rightarrow \mathbb {R}_{\ge 0}$ for each $C \in \mathbb {R}_{\ge 0}$. In general, the function $\varphi$ can oscillate wildly. Let us consider, for example, $\varphi$ when $\theta _1$ is given as above. In this case,

26 $$\begin{aligned} \varphi (x_1) = \frac{ C^{2\mathrm{mod}(x_1,2) - 1}\prod _{i=0}^{\lfloor x_1/2 \rfloor - 1}\theta _1(x_1 - 2i)}{ \prod _{i=0}^{\lfloor (x_1-1)/2 \rfloor - 1}\theta _1(x_1 - 2i - 1)}. \end{aligned}$$

**Fig. 2:**
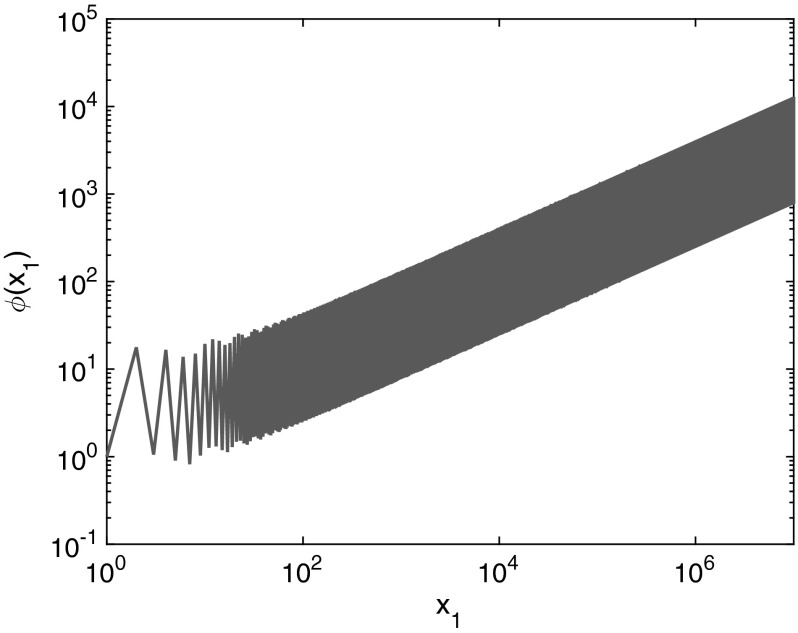
(Color figure online) $\varphi$ as given in (25) with $C=1$.

**Fig. 3:**
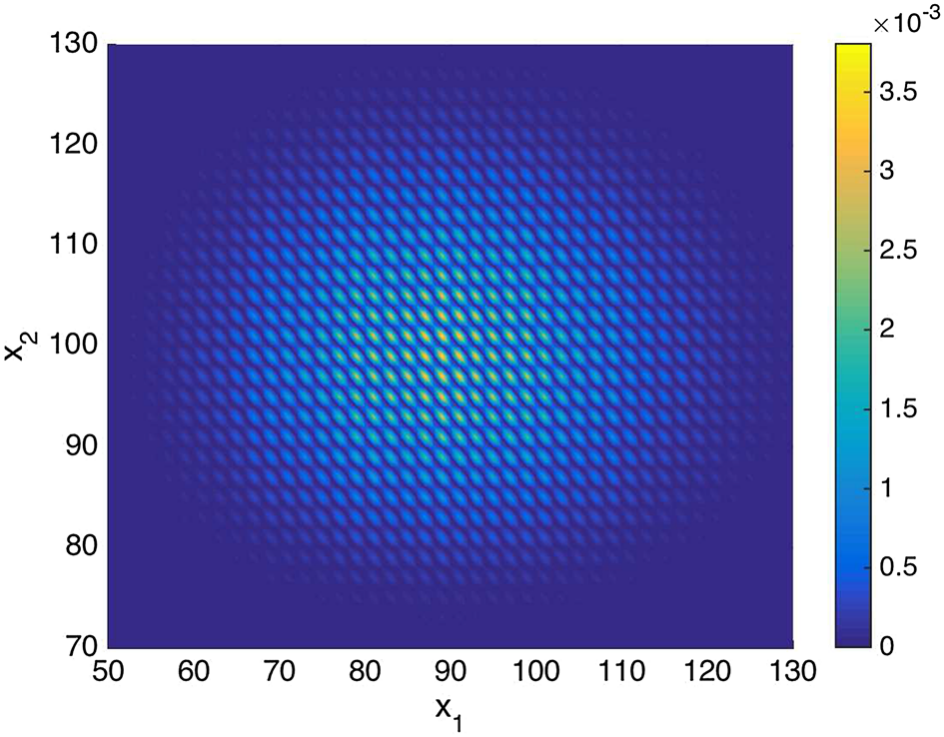
(Color figure online) Stationary distribution (27) of the system (25) with parameters given by (28), with the assumption that the initial value of $x_1+x_2$ is even. The normalization constant $\Gamma$ was approximated by summing the value of all states $x \in \{0,1,\ldots ,1000\} \times \{0,1,\ldots ,1000\}$.

Figure 2 demonstrates how $\varphi (x_1)$ and the amplitude of the oscillations grow with $x_i$, for the case $C=1$. It is clear that this function does not represent any physical reaction rate arising from chemistry, but we can still write down the invariant distribution of the system (25).

The complex balance equilibrium of the associated mass action kinetics system to (25) is given by $(c_1,c_2) = \left( \sqrt{\frac{k_2}{k_1}}, \frac{k_3}{k_4}\sqrt{\frac{k_2}{k_1}} \right)$. Therefore, the stationary distribution is given by:

27 $$\begin{aligned} \pi (x) = \frac{1}{\Gamma } \frac{c_1^{x_1}}{\prod _{i=1}^{x_1} \varphi (x_1)} \frac{c_2^{x_2}}{x_2!},\quad x \in \{(x_1,x_2)\ |\ \mathrm{mod}(x_1+x_2,2) = a\} \quad \mathrm{for} \quad a \in \{0,1\},\nonumber \\ \end{aligned}$$

where $\Gamma$ is a normalizing constant and $\varphi$ is given by (26) with $C=1$. Note that the value of *a* here dictates the oddness or evenness of the quantity $x_1+x_2$, which is preserved by each of the reactions.

Figure 3 shows the invariant distribution (27) of the system (25) with an even initial value of $x_1 + x_2$, and for the following parameters:

28 $$\begin{aligned} k_1 = 1, \qquad k_2 = 10^2, \qquad k_3 = 10, \qquad k_4 = 1. \end{aligned}$$

The normalization constant was approximated by summing the values of all states $x \in \{0,1,\ldots ,1000\} \times \{0,1,\ldots ,1000\}$.

## Conclusions

In this paper, we provided the stationary distributions for the class of stochastically modeled reaction networks with non-mass action kinetics that satisfy our Assumption 1 and that admit a complex-balanced equilibrium when modeled deterministically with mass action kinetics. Similarly to the results of Anderson et al. (2010), we showed that the stationary distributions are of product form.

We motivated the need for such results through consideration of modern averaging techniques. In particular, Theorem 2 significantly reduces the computational cost of finding the invariant distribution of most fast subsystems in a multiscale setting, therefore making accurate approximations very cheap to compute. This in turn opens up more possibilities, for example approximation of likelihoods via multiscale reductions in the context of parameter inference for biochemical networks.

## Appendix: Terminology and Results from Chemical Reaction Network Theory

For each reaction network, $\{\mathcal {S},\mathcal {C},\mathcal {R}\}$, there is a unique directed graph constructed in the following manner. The nodes of the graph are the complexes, $\mathcal {C}$. A directed edge is then placed from complex $\nu _k$ to complex $\nu _k'$ if $\nu _k \rightarrow \nu _k' \in \mathcal {R}$. Each connected component of the resulting graph is termed a *linkage class*. We denote the number of linkage classes by $\ell$. For example, in the reaction network (9) there are two linkage classes.

### Definition 3

A chemical reaction network, $\{\mathcal {S},\mathcal {C},\mathcal {R}\}$, is called *weakly reversible* if each linkage classes is strongly connected. A network is called *reversible* if $\nu _k' \rightarrow \nu _k \in \mathcal {R}$ whenever $\nu _k \rightarrow \nu _k' \in \mathcal {R}$.

Note that a network is weakly reversible if and only if for any reaction $\nu _k \rightarrow \nu _k'$, there is a sequence of directed reactions beginning with $\nu _k'$ as a source complex and ending with $\nu _k$ as a product complex. That is, there exist complexes $\nu _1,\dots ,\nu _r$ such that $\nu _k' \rightarrow \nu _1, \nu _1 \rightarrow \nu _2, \dots , \nu _r \rightarrow \nu _k \in \mathcal {R}$.

### Definition 4

$S ={\mathrm{span}}_{\{\nu _k \rightarrow \nu _k' \in \mathcal {R}\}}\{\nu _k' - \nu _k\}$ is the *stoichiometric subspace* of the network. For $z \in {\mathbb {R}}^d$, we say $z + S$ and $(z + S) \cap {\mathbb {R}}^d_{>0}$ are the *stoichiometric compatibility classes* and *positive stoichiometric compatibility classes* of the network, respectively. Denote $\hbox {dim}(S) = s$.

The final definition is that of the *deficiency* of a network (Feinberg 1987).

### Definition 5

The *deficiency* of a chemical reaction network, $\{\mathcal {S},\mathcal {C},\mathcal {R}\}$, is $\delta = |\mathcal {C}| - \ell - s$, where $|\mathcal {C}|$ is the number of complexes, $\ell$ is the number of linkage classes of the network graph, and *s* is the dimension of the stoichiometric subspace of the network.

We state a classical result that can be found in Feinberg (1972, 1979, 1987), Gunawardena (2003), Horn (1972), which relates networks that are weakly reversible and have a deficiency of zero to those that admit complex-balanced equilibria.

### Theorem 3

If the reaction network $\{\mathcal {S},\mathcal {C},\mathcal {R}\}$ is weakly reversible and has a deficiency of zero, then for any choice of rate constants $\kappa _k$ the deterministic mass action system admits a complex-balanced equilibrium, *c*, satisfying (2). Moreover, within each positive stoichiometric compatibility class, there is precisely one equilibrium, and it is complex balanced.
